# Long term effects of smoking cessation in hospitalized schizophrenia patients

**DOI:** 10.1186/s12888-017-1250-1

**Published:** 2017-03-07

**Authors:** Masatoshi Miyauchi, Ikuko Kishida, Akira Suda, Yohko Shiraishi, Mami Fujibayashi, Masataka Taguri, Chie Ishii, Norio Ishii, Toshio Moritani, Yoshio Hirayasu

**Affiliations:** 1 Department of Psychiatry, Yokohama City University School of Medicine, 3-9 Fukuura, Kanazawa-ku, Yokohama, Kanagawa 236-0004 Japan; 2 Fujisawa Hospital, 383 Kotsuka, Fujisawa, Kanagawa 251-8530 Japan; 3 Clinical Laboratory Department, Yokohama City University Hospital, 3-9 Fukuura, Kanazawa-ku, Yokohama, Kanagawa 236-0004 Japan; 4 The Division of Physical and Health Education, Setsunan University, 17-8 Ikedanakamachi, Neyagawa, Osaka 572-8508 Japan; 5 Department of Biostatistics, Yokohama City University School of Medicine, 3-9 Fukuura, Kanazawa-ku, Yokohama, Kanagawa 236-0004 Japan; 6 Faculty of General Education, Kyoto Sangyo University, Kamo-motoyama, Kita-Ku, Kyoto, 606-8555 Japan

**Keywords:** Autonomic nervous system, Patient with schizophrenia, Smoking cessation, Antipsychotics, Antiparkinsonian drug, Cholesterol, Body mass index

## Abstract

**Background:**

The prevalence of smoking in patients with schizophrenia is higher than that in the general population and is an important medical issue. Short-term smoking cessation tends to worsen psychiatric symptoms in patients with schizophrenia but decreases sympathetic nervous system activity and improves plasma cholesterol levels in healthy people. Few studies have assessed the long-term effects of smoking cessation in patients with schizophrenia.

**Methods:**

Subjects were 70 Japanese patients with schizophrenia (38 smokers, 32 non-smokers). We compared the following clinical parameters between the two groups at baseline (before smoking cessation) and in each group separately between baseline and at three years after smoking cessation: autonomic nervous system activity, plasma cholesterol levels, body weight, drug therapy, and Global Assessment of Functioning scores. We also compared the mean changes in clinical parameters throughout this study between the groups at both time points. Autonomic nervous system activity was assessed by power spectral analysis of heart rate variability.

**Results:**

Parasympathetic nervous system activity and the doses of antiparkinsonian drugs in smokers were significantly higher than those in non-smokers at baseline. Smoking cessation was associated with significantly decreased sympathetic nervous system activity and decreased doses of antipsychotics and antiparkinsonian drugs at three years after smoking cessation. However, there was no significant difference in the mean change in clinical factors scores, except for Global Assessment of Functioning scores, between smokers and non-smokers at three years after smoking cessation.

**Conclusions:**

Our results suggest that smoking reduces both autonomic nervous system activity and the effectiveness of drug therapy with antipsychotics and antiparkinsonian drugs in patients with schizophrenia, but that both factors could be ameliorated over the long term by smoking cessation. Taken together with the findings of previous studies, smoking cessation in patients with schizophrenia has many long-term positive physiological effects.

## Background

The prevalence of smoking in patients with schizophrenia is at least two to three times higher than that in the general population [1–3]. Patients with schizophrenia experience greater difficulties in smoking cessation compared with the general population [1–3]. Given the problems associated with smoking, this habit among patients with schizophrenia is of clinical significance. Smoking cessation has various positive physiological effects in healthy people, including lower serum cholesterol levels and decreased sympathetic nervous system activity [4–9]; increased sympathetic nervous activity increases the risk of cardiac disease, such as atrial and ventricular arrhythmias, cardiac arrest, and acute myocardial infarction [10–13]. However, as a negative physiological effect, a previous study reported that smoking cessation for one year resulted in weight gain [14].

Several studies have investigated the effects of smoking and smoking cessation in patients with schizophrenia. One study showed an association between smoking and high serum lipid profiles [15], and another found that smoking cessation for one year was associated with worsening of Global Assessment of Functioning (GAF) scores [16]. However, few studies have assessed the long-term effects of smoking cessation in these patients. The present study aimed to address this gap by investigating the long-term effects of smoking cessation in patients with schizophrenia, such as the effects on serum cholesterol levels, body weight, drug therapy, GAF scores, and autonomic nervous system (ANS) activity.

## Methods

### Subjects

This study involved 38 smokers (7 men, 31 women; 57.9 ± 12.5 years) and 32 non-smokers (11 men, 21 women; 54.3 ± 13.6 years) with schizophrenia diagnosed according to the Diagnostic and Statistical Manual of Mental Disorders, fourth edition (DSM-IV), by a psychiatrist. They were all inpatients at a psychiatric hospital during the study period. Exclusion criteria were (1) onset of chronic medical conditions (e.g., diabetes, respiratory disease, cardiac disease, or ANS disorders) at any point during the study and (2) cardiovascular disease, arrhythmia, and long QT syndrome as determined in all patients by chest radiography and electrocardiography. Regular blood tests, chest radiography and electrocardiography were performed once a year and subjects were evaluated by a psychiatrist regularly throughout the study period. All 38 smokers were habitual smokers, with a Tobacco Dependence Screener (TDS) score of > 5 points (mean score 5.71 ± 0.65). The TDS is a self-administered questionnaire for assessing nicotine dependence as defined by the 10th revision of the International Statistical Classification of Diseases and Related Health Problems and DSM-IV [17], the cutoff score for TDS is 5 points for diagnosis according to ICD-10 [17]. The 32 non-smokers had never smoked before. Based on the stipulation of the hospital’s own smoke-free policy instituted in 2010, the 38 smokers quit smoking. After instituting the policy, we assessed the subjects over the subsequent three-year period. None of the 38 smokers received any medication or behavioral therapy for smoking cessation.

The clinical parameters studied at baseline (before smoking cessation in smokers) and at three years after smoking cessation were all related to the effects of smoking. These parameters included body mass index (BMI) and serum levels of albumin (ALB), blood sugar (BS), low-density lipoprotein cholesterol (LDL-C), total cholesterol (TC), triglycerides (TG), and high-density lipoprotein cholesterol (HDL-C). In addition, subjects were evaluated and assigned GAF scores according to DSM-IV as assessed by a psychiatrist at baseline and at three years after smoking cessation. We also investigated the use of antipsychotics, antiparkinsonian drugs, and anxiolytics, namely chlorpromazine, biperiden, and diazepam, respectively. The required therapeutic doses of these drugs were calculated for each patient according to the standard equivalent conversions [18].

The study protocol was approved by the Institutional Review Board of Seishinkai Fujisawa Hospital, and the study was performed in accordance with the Declaration of Helsinki. All patients provided written informed consent after receiving detailed information regarding the study.

### R-R interval power spectral analysis

ANS activity was quantified using heart rate variability (HRV) power spectral analysis. The technique employed in the present investigation for detecting the three major spectral components of HRV has been previously applied under diverse psychophysiological conditions in basic medical science and clinical research. The validity, reliability, and practicability of this technique have been well documented [19–21]. HRV power spectral analysis decomposes a series of sequential R–R intervals obtained from electrocardiography (ECG) for 5 min into a sum of sinusoidal functions of different amplitudes and frequencies by the Fast Fourier transform algorithm [22–27]. The spectral powers in the frequency domain were quantified by integrating the area under the curve for the following respective bandwidth measurements: the low-frequency (LF; 0.03–0.15 Hz) component of HRV representing both sympathetic and parasympathetic activity; the high-frequency (HF; 0.15–0.4 Hz) component associated almost entirely with parasympathetic nerve activity; and total power (TP; 0.03–0.4 Hz) representing overall cardiac ANS activity [24–26].

### Statistical analysis

The clinical parameters (HRV; BMI; plasma levels of ALB, TP, BS, LDL-C, TC, TG, and HDL-C; doses of antipsychotics, antiparkinsonian drugs, and anxiolytic drugs; and GAF scores) were compared between smokers and non-smokers at baseline using Student’s unpaired *t*-test, and in each group separately between baseline and at three years after smoking cessation using Wilcoxon’s signed-rank test. Additionally, to evaluate the effects of smoking cessation, mean changes in the clinical parameters were compared between the two groups at both time points using the unpaired *t*-test. Total power, LF, and HF data were logarithmically transformed using Log10 to obtain normally distributed data. All statistical analyses were performed with SPSS for Windows Version 21.0 (IBM Corp., Armonk, NY). A *p* value of < 0.05 was considered statistically significant.

## Results

Table 1 shows the baseline demographic and clinical parameters for all subjects. The 38 smokers comprised 7 men and 31 women, with a mean age (± standard deviation) of 58.5 ± 2.3 years; the 32 non-smokers comprised 11 men and 21 women with a mean age of 54.3 ± 13.6 years. Mean duration of illness was 33.2 ± 14.5 years and 32.7 ± 13.5 years, respectively. Compared with non-smokers, smokers had a significantly higher HF component and significantly higher doses of antiparkinsonian drugs (both *p* < 0.05).

**Table 1:** Clinical parameters between smokers and non-smokers at baseline (before cessation of smoking)

	Smokers	Non-smokers	*P value*
Number	38	32	
Sex	7/31	11/21	
Age (years)	57.9 ± 12.5	54.3 ± 13.6	0.241
Duration of illness	33.2 ± 14.5	32.7 ± 13.5	0.876
ln total^1^	4.5 ± 1.2	3.9 ± 1.2	0.071
ln LF^1^	3.8 ± 1.3	3.2 ± 1.4	0.163
ln HF^1^	3.8 ± 1.2	3.1 ± 1.3	0.016^*^
BMI (kg/m^2^)	22.7 ± 4.0	21.7 ± 4.0	0.276
ALB (g/dL)	4.0 ± 0.3	4.0 ± 0.4	0.204
BS (mg/dL)	89.7 ± 17.4	87.5 ± 15.5	0.655
LDL-C (mg/dL)	119.0 ± 33.2	108.4 ± 29.6	0.163
TC (mg/dL)	200.4 ± 42.2	183.9 ± 32.0	0.065
TG (mg/dL)	118.9 ± 91.1	86.0 ± 43.2	0.073
HDL-C (mg/dL)	60.4 ± 16.6	55.9 ± 13.2	0.222
CPZeq^*2^ (mg)	1229 ± 750	1162 ± 792	0.717
BPDeq^*2^ (mg)	2.9 ± 1.9	1.6 ± 1.6	0.003^*^
DZPeq^*2^ (mg)	10.1 ± 6.9	9.3 ± 7.4	0.653
GAF score	28.9 ± 3.1	29.2 ± 2.2	0.682

Data are presented as means ± standard deviation

^*^Significant difference (*p* < 0.05)

^1^Logarithmic conversion was performed

^2^Daily dose of antipsychotic was converted to the approximate respective equivalent dose

*LF* Low Frequency, *HF* High frequency, *BMI* Body mass index, *ALB* Serum albumin, *BS* Blood sugar, LDL-C Low-density lipoprotein cholesterol, *TC* Total cholesterol, *TG* Triglycerides, *HDL-C* High-density lipoprotein cholesterol, *CPZeq* Equivalent conversions of chlorpromazine, *BDPeq* Equivalent conversions of biperiden, *DZP* Equivalent conversions of diazepam, *GAF* Global Assessment of Functioning

Table 2 shows the clinical parameters at baseline and at three years after smoking cessation in both groups as well as the mean changes at both time points. At three years after smoking cessation compared with at baseline, smokers had a significantly lower LF component and significantly decreased doses of antipsychotics and antiparkinsonian drugs (both *p* < 0.05). Most subjects were receiving >1 antipsychotic. BMI was significantly decreased and serum HDL-C was significantly increased in both groups (both *p* < 0.05). Among the mean changes in clinical parameters analyzed, only GAF score was significantly different between smokers and non-smokers, showing significant worsening in smokers (*p* < 0.05).

**Table 2:** Changes in clinical parameters between baseline and three years later in smokers and in non-smokers

	Smokers				Non-smokers				
	*n* = 38				*n* = 32				
	Baseline	Three years	Changes	*p*	Baseline	Three years	Changes	*P*	*Comparison*
ln total^1^	4.5 ± 1.2	4.0 ± 1.7	−0.50 ± 1.57	0.061	3.9 ± 1.2	3.9 ± 1.4	−0.11 ± 0.95	0.681	0.218
ln LF^1^	3.8 ± 1.3	3.0 ± 1.8	−0.79 ± 1.73	0.049^*^	3.2 ± 1.4	3.2 ± 1.5	−0.09 ± 0.96	0.715	0.133
ln HF^1^	3.8 ± 1.2	3.4 ± 1.7	−0.44 ± 1.67	0.136	3.1 ± 1.3	3.1 ± 1.5	−0.85 ± 1.17	0.915	0.304
BMI (kg/m^2^)	22.7 ± 4.0	21.1 ± 3.6	−1.56 ± 3.64	0.020^*^	21.7 ± 4.0	20.8 ± 3.9	−0.92 ± 2.17	0.022^*^	0.373
ALB (g/dL)	4.0 ± 0.3	3.9 ± 0.4	−0.03 ± 0.38	0.564	4.0 ± 0.4	3.9 ± 0.4	−0.15 ± 0.46	0.076	0.240
BS (mg/dL)	89.7 ± 17.4	87.6 ± 14.6	−1.7 ± 18.6	0.568	87.5 ± 15.5	87.7 ± 14.1	0.16 ± 16.2	0.957	0.654
LDL-C (mg/dL)	119.0 ± 33.2	115.6 ± 36.2	−2.3 ± 39.0	0.717	108.4 ± 29.6	110.2 ± 27.3	1.7 ± 29.2	0.740	0.631
TC (mg/dL)	200.4 ± 42.2	190.4 ± 42.2	−10.4 ± 52.1	0.291	183.9 ± 32.0	186.3 ± 35.1	2.4 ± 27.2	0.620	0.421
TG (mg/dL)	118.9 ± 91.1	100.0 ± 48.0	−18.9 ± 93.1	0.212	86.0 ± 43.2	96.1 ± 40.8	10.2 ± 46.2	0.223	0.096
HDL-C (mg/dL)	60.4 ± 16.6	70.3 ± 18.0	9.92 ± 18.2	0.002^*^	55.9 ± 13.2	64.0 ± 18.9	8.1 ± 13.5	0.002^*^	0.635
CPZeq^2^ (mg)	1229 ± 750	1008 ± 646	−221 ± 576	0.031^*^	1162 ± 792	1075 ± 810	−86.7 ± 489	0.323	0.302
BPDeq^2^ (mg)	2.9 ± 1.9	2.3 ± 1.7	−0.63 ± 1.7	0.018^*^	1.6 ± 1.6	1.7 ± 1.7	0.15 ± 1.8	0.623	0.061
DZPeq^2^ (mg)	10.1 ± 6.9	8.6 ± 6.9	−1.5 ± 6.4	0.190	9.3 ± 7.4	9.1 ± 7.4	−0.12 ± 6.6	0.914	0.379
GAF score	28.9 ± 3.1	27.9 ± 4.1	−1.1 ± 3.1	0.444	29.2 ± 2.2	29.4 ± 2.1	0.15 ± 1.5	0.572	0.040^*^

Data are presented as means ± standard deviation

*Significant difference *p* < 0.05

^1^Logarithmic conversion was performed

^2^The daily dose of antipsychotics was converted to the approximate respective equivalent dose

*LF* Low frequency, *HF* High frequency, *BMI* Body mass index, *ALB* Albumin, *BS* Blood sugar, *LDL-C* Serum low-density lipoprotein cholesterol, *TC* Total cholesterol, *TG* Triglycerides, *HDL-C* High-density lipoprotein cholesterol, *CPZeq* Equivalent conversions of chlorpromazine, *BDPeq* Equivalent conversions of biperiden, *DZP* Equivalent conversions of diazepam, *GAF* Global Assessment of Functioning

## Discussion

### Comparison of clinical parameters between smokers and non-smokers at baseline

Our results showed that smokers had a higher HF component than non-smokers, suggesting increased parasympathetic nervous system activity in smokers with schizophrenia. This finding differs from that in healthy subjects, in which smokers had higher sympathetic nervous system activity than non-smokers [4–6], and it supports the previous findings of abnormal autonomic nervous system activity in patients with schizophrenia compared with healthy people [28, 29]. The required doses of antiparkinsonian drugs at baseline were significantly higher in smokers than in non-smokers. We suggest that this finding is accounted for by higher required doses of antipsychotic drugs in smokers compared with non-smokers.

### Clinical parameters at baseline and at three years after smoking cessation

Our results demonstrate that long-term smoking cessation in smokers with schizophrenia was associated with a decreased LF component, which might indicate reduced sympathetic nervous system activity resulting from smoking cessation. Smoking stimulates nicotinic acetylcholine receptors in the central nervous system and increases inflammation and oxidative stress in the lungs, leading to increased sympathetic activity via activation of afferent neurons in the lungs [4]. Thus, our finding suggesting that smoking cessation improved sympathetic nervous system activity seems reasonable. In contrast, the HF component showed no significant change after long-term smoking cessation in our patient population, which is inconsistent with the findings of previous studies involving healthy subjects [4, 30, 31]. Given that patients with schizophrenia have much lower parasympathetic nervous system activity than healthy people [28, 29], our findings seem to be influenced more by this much lower activity than by smoking.

In this study, the required doses of antipsychotics and antiparkinsonian drugs of the smokers were significantly decreased after smoking cessation, but the doses of anxiolytic drugs were not affected. Because smoking increases the activity of cytochrome P450 isoenzyme 1A2 (CYP1A2) and UDP-glucuronosyltransferases (UGT), which are responsible for drug metabolism, the metabolism of antipsychotics is consequently influenced by smoking [32]. Smoking cessation therefore results in decreased metabolism of antipsychotics via CYP1A2 and UGT and thus in the required dose of antipsychotics. This is supported by the finding that, after smoking cessation, dopamine levels were increased following a decrease in the stimulation of nicotinic acetylcholine receptors [33]. Our results also suggest that smoking cessation in the long term decreased the required dose of antiparkinsonian drugs, as a consequence of taking lower doses of antipsychotics after smoking cessation.

In this study, smoking cessation in inpatients with schizophrenia lowered serum HDL-C, similar to findings in healthy individuals [7, 9]. Moreover, we found that BMI was decreased after smoking cessation in these inpatients, although this finding conflicts with that of a meta-analysis reporting that people experience an increase in body weight after smoking cessation because of improved appetite [14]. This discrepancy might be because our subjects were inpatients and received hospital meals monitored by a nutritionist every day throughout this study. In smokers with schizophrenia, some antipsychotics cause substantial weight gain and therefore the reduced required dose of antipsychotics that we observed may have resulted in the decreased BMI observed after smoking cessation. Moreover, because patients with schizophrenia usually gain weight after smoking cessation, we educated all subjects to avoid weight gain. Further research is needed to confirm this speculation. Our results showed that both smokers and non-smokers had lower HDL-C and BMI in the long term and this may have been influenced by factors other than smoking cessation.

### Mean changes in clinical parameters at three years among smokers and non-smokers

Our results showed a significant difference in GAF scores between smokers and non-smokers. A previous study revealed that smoking cessation worsened psychotic symptoms and increased the propensity for violence in psychiatric patients [34]. Moreover, another study reported worsened GAF scores at one year after smoking cessation [16], which is consistent with our long-term assessment results [16]. However, we found no significant difference in clinical factors that could explain the difference in GAF score. Further study is needed to assess the effects on psychotic symptoms in the long term.

### Limitations of the study

Our study has some limitations. First, it was conducted at a single institution and the number of subjects was relatively small. This does not adequately reflect significant differences in the mean changes in clinical parameters between baseline and at three years among smokers and non-smokers. Second, we did not investigate patients with schizophrenia who continued to smoke and we did not consider the effect of sex on cholesterol levels and ANS activity. Third, our assessment of whether subjects smoked or not was subjective, not objective. Fourth, we did not analyze the long-term effects of smoking cessation in the short term and whether smoking cessation affected psychiatric symptoms such as indicated by the Positive and Negative Syndrome Scale (PANSS). Fifth, we did not determine the serum concentrations of the antipsychotics and did not consider the metabolism of individual antipsychotics directly influenced by smoking, including olanzapine and haloperidol. Sixth, we did not take multiple testing into account because this was exploratory research. Finally, most of our subjects were patients with schizophrenia who had been hospitalized for a long time and these results do not therefore apply to all patients with schizophrenia. Further study with a larger sample size and other subjects such as outpatients with schizophrenia is needed to verify our results.

## Conclusions

Our findings suggest that long-term effects of smoking cessation among inpatients with schizophrenia were not only associated with decreased required doses of antipsychotics and antiparkinsonian drugs but also with improved ANS activity. Further studies are needed to clarify whether smoking cessation leads to long-term changes in the health of patients with schizophrenia.

## Abbreviations

ALB: Serum albumin
ANS: Autonomic nervous system (ANS)
BMI: Body mass index
BPDeq: Equivalent conversions of biperiden
BS: Blood sugar
CPZeq: Equivalent conversions of chlorpromazine
DZPeq: Equivalent conversions of diazepam
GAF: Global Assessment of Functioning
HDL-C: High-density lipoprotein cholesterol
HF: High frequency
HRV: Heart rate variability
LDL-C: Low-density lipoprotein cholesterol
LF: Low frequency
TC: Total cholesterol
TG: Triglycerides
